# Effects of an Exercise Program on Brain Health Outcomes for Children With Overweight or Obesity: The ActiveBrains Randomized Clinical Trial

**DOI:** 10.1001/jamanetworkopen.2022.27893

**Published:** 2022-08-30

**Authors:** Francisco B. Ortega, Jose Mora-Gonzalez, Cristina Cadenas-Sanchez, Irene Esteban-Cornejo, Jairo H. Migueles, Patricio Solis-Urra, Juan Verdejo-Román, María Rodriguez-Ayllon, Pablo Molina-Garcia, Jonatan R. Ruiz, Vicente Martinez-Vizcaino, Charles H. Hillman, Kirk I. Erickson, Arthur F. Kramer, Idoia Labayen, Andrés Catena

**Affiliations:** 1 PROFITH “PROmoting FITness and Health Through Physical Activity” Research Group, Sport and Health University Research Institute (iMUDS), Department of Physical Education and Sports, Faculty of Sport Sciences, University of Granada, Granada, Spain; 2 Faculty of Sport and Health Sciences, University of Jyväskylä, Jyväskylä, Finland; 3 Department of Biosciences and Nutrition, Karolinska Institutet, Huddinge, Sweden; 4 Department of Health, Medicine and Caring Sciences, Linköping University, Linköping, Sweden; 5 Faculty of Education and Social Sciences, Universidad Andres Bello, Viña del Mar, Chile; 6 Department of Personality, Assessment and Psychological Treatment and Mind, Brain, and Behavior Research Center (CIMCYC), University of Granada, Granada, Spain; 7 Laboratory of Cognitive and Computational Neuroscience (UCM-UPM), Centre for Biomedical Technology (CTB), Madrid, Spain; 8 Department of Epidemiology, Erasmus MC University Medical Center, Rotterdam, the Netherlands; 9 Biohealth Research Institute, Physical Medicine and Rehabilitation Service, Virgen de las Nieves University Hospital, Granada, Spain; 10 Instituto de Investigación Biosanitaria, ibs.Granada, Granada, Spain; 11 Health and Social Research Center, Universidad de Castilla La Mancha, Cuenca, Spain; 12 Faculty of Health Sciences, Universidad Autónoma de Chile, Talca, Chile; 13 Department of Psychology, Northeastern University, Boston, Massachusetts; 14 Department of Physical Therapy, Movement and Rehabilitation Sciences, Northeastern University, Boston, Massachusetts; 15 Brain Aging & Cognitive Health Lab, Department of Psychology, University of Pittsburgh, Pittsburgh, Pennsylvania; 16 College of Science, Health, Engineering, and Education, Murdoch University, Perth, Western Australia; 17 Beckman Institute, University of Illinois at Urbana-Champaign, Champaign; 18 Department of Health Sciences and Institute for Innovation & Sustainable Food Chain Development (IS-FOOD), Public University of Navarra, Pamplona, Spain; 19 IdiSNA, Navarra Institute for Health Research, Pamplona, Spain; 20 School of Psychology, University of Granada, Granada, Spain

## Abstract

**Importance:**

Pediatric overweight and obesity are highly prevalent across the world, with implications for poorer cognitive and brain health. Exercise might potentially attenuate these adverse consequences.

**Objectives:**

To investigate the effects of an exercise program on brain health indicators, including intelligence, executive function, academic performance, and brain outcomes, among children with overweight or obesity and to explore potential mediators and moderators of the main effects of exercise.

**Design, Setting, and Participants:**

All preexercise and postexercise data for this 20-week randomized clinical trial of 109 children aged 8 to 11 years with overweight or obesity were collected from November 21, 2014, to June 30, 2016, with neuroimaging data processing and analyses conducted between June 1, 2017, and December 20, 2021. All 109 children were included in the intention-to-treat analyses; 90 children (82.6%) completed the postexercise evaluation and attended 70% or more of the recommended exercise sessions and were included in per-protocol analyses.

**Interventions:**

All participants received lifestyle recommendations. The control group continued their usual routines, whereas the exercise group attended a minimum of 3 supervised 90-minute sessions per week in an out-of-school setting.

**Main Outcomes and Measures:**

Intelligence, executive function (cognitive flexibility, inhibition, and working memory), and academic performance were assessed with standardized tests, and hippocampal volume was measured with magnetic resonance imaging.

**Results:**

The 109 participants included 45 girls (41.3%); participants had a mean (SD) body mass index of 26.8 (3.6) and a mean (SD) age of 10.0 (1.1) years at baseline. In per-protocol analyses, the exercise intervention improved crystallized intelligence, with the exercise group improving from before exercise to after exercise (mean *z* score, 0.62 [95% CI, 0.44-0.80]) compared with the control group (mean *z* score, –0.10 [95% CI, –0.28 to 0.09]; difference between groups, 0.72 SDs [95% CI, 0.46-0.97]; *P* < .001). Total intelligence also improved significantly more in the exercise group (mean *z* score, 0.69 [95% CI, 0.48-0.89]) than in the control group (mean *z* score, 0.07 [95% CI, –0.14 to 0.28]; difference between groups, 0.62 SDs [95% CI, 0.31-0.91]; *P* < .001). Exercise also positively affected a composite score of cognitive flexibility (mean *z* score: exercise group, 0.25 [95% CI, 0.05-0.44]; control group, –0.17 [95% CI, –0.39 to 0.04]; difference between groups, 0.42 SDs [95% CI, 0.13-0.71]; *P* = .005). These main effects were consistent in intention-to-treat analyses and after multiple-testing correction. There was a positive, small-magnitude effect of exercise on total academic performance (mean *z* score: exercise group, 0.31 [95% CI, 0.18-0.44]; control group, 0.10 [95% CI, –0.04 to 0.24]; difference between groups, 0.21 SDs [95% CI, 0.01-0.40]; *P* = .03), which was partially mediated by cognitive flexibility. Inhibition, working memory, hippocampal volume, and other brain magnetic resonance imaging outcomes studied were not affected by the exercise program. The intervention increased cardiorespiratory fitness performance as indicated by longer treadmill time to exhaustion (mean *z* score: exercise group, 0.54 [95% CI, 0.27-0.82]; control group, 0.13 [95% CI, –0.16 to 0.41]; difference between groups, 0.42 SDs [95% CI, 0.01-0.82]; *P* = .04), and these changes in fitness mediated some of the effects (small percentage of mediation [approximately 10%-20%]). The effects of exercise were overall consistent across the moderators tested, except for larger improvements in intelligence among boys compared with girls.

**Conclusions and Relevance:**

In this randomized clinical trial, exercise positively affected intelligence and cognitive flexibility during development among children with overweight or obesity. However, the structural and functional brain changes responsible for these improvements were not identified.

**Trial Registration:**

ClinicalTrials.gov Identifier: NCT02295072

## Key Points

**Question:**

Can an exercise intervention of aerobic plus resistance training improve cognitive and brain health outcomes for children with overweight or obesity?

**Findings:**

In this randomized clinical trial of 109 participants, exercise significantly improved intelligence and cognitive flexibility among preadolescent children with overweight or obesity. There was also a positive, smaller-magnitude significant effect of exercise on academic performance but no significant effect on inhibition and working memory or on structural and functional brain outcomes studied.

**Meaning:**

This study suggests that exercise can positively affect intelligence and cognitive flexibility during a sensitive period of brain development in childhood and, to a smaller extent, academic performance, indicating that an active lifestyle before puberty may lead to more successful life trajectories.

## Teaser

This randomized clinical trial investigates the effects of exercise on brain health indicators, including intelligence, executive function, academic performance, and brain outcomes, among children with overweight or obesity and explores mediators and moderators of the main effects of exercise.

## Introduction

The prevalence of overweight and obesity among youths has more than quadrupled worldwide from 1975 to 2016 (from 4% to 18%).^1^ Evidence suggests that obesity might negatively affect brain health (ie, cognitive and brain development).^2,3,4^ It is therefore necessary to identify effective strategies to attenuate these adverse consequences. Physical exercise is a candidate to produce such positive stimuli because it provides multisystemic benefits to human organs, including the brain.^5,6^ Existing exercise-based interventions have mostly targeted executive functions and other dimensions of cognition (eg, processing speed and language),^7,8,9^ yet, to our knowledge, evidence regarding the effect of exercise on intelligence and its components (ie, crystallized intelligence and fluid intelligence)^10^ is lacking. Against traditional beliefs, the notion that intelligence is “malleable” despite its high heritability is gaining support,^11^ yet more research is warranted.

Although most previous studies focused on behavioral outcomes (eg, executive function and other dimensions of cognition), only a few randomized clinical trials (RCTs) for children have investigated the effects of exercise on brain structure and function.^12,13,14,15,16,17,18,19,20^ There is a need for high-quality RCTs that combine behavioral and brain imaging outcomes, as well as a better characterization of the exercise dose administered in the interventions.^21,22^ Moreover, previous studies of animals^23^ and older adults^23,24,25^ have pointed to hippocampal volume as a critical brain outcome affected by exercise. Although the hippocampus is not a brain region directly associated with intelligence, it is a central hub in networks that support executive function and memory. The effects of exercise on this brain region during a period of brain growth remain underinvestigated, to our knowledge. Furthermore, a comprehensive investigation, including a broader set of magnetic resonance imaging (MRI) outcomes, is needed to understand the overall effect of exercise on brain structure and function.

The ActiveBrains RCT^26^ included a broad set of both behavioral and brain MRI outcomes and was designed to test the effects of exercise on brain health among children with overweight or obesity. Our primary aim (a priori planned) was to investigate the effects of a 20-week exercise program on behavioral outcomes, including intelligence, executive function (ie, cognitive flexibility, inhibition, and working memory), and academic performance as well as on hippocampal volume as a primary region of interest in children with overweight or obesity.

In secondary analyses (a posteriori planned), we explored potential mediators and moderators of the main exercise effects observed in this intervention. First, we investigated cardiorespiratory fitness (CRF) as the main candidate mediator,^27,28,29,30,31,32,33,34,35,36,37,38^ and we explored other specific brain regions of interest (eg, the prefrontal cortex because of its relationship with intelligence and cognitive flexibility^39,40,41^) and broader brain structural and functional changes (hypothesis-free analyses) as potential mediators. Second, we tested potential moderators (sex, age, maturation, socioeconomic status, and baseline performance) of the intervention effects.^42^ Third, we interrogated potential compensatory and contamination effects on daily activity levels, which were assessed with accelerometers. Fourth, we analyzed the exercise dose (ie, the actual volume and intensity of the intervention, assessed via heart rate monitoring) because this dose might have a direct effect on the magnitude of intervention effects.

## Methods

A brief description of the material and methods is discussed. The trial protocol and statistical analysis plan are provided in Supplement 1. All methodological details are provided in the eMethods in Supplement 2.

### Study Design and Participants

**Figure 1.**
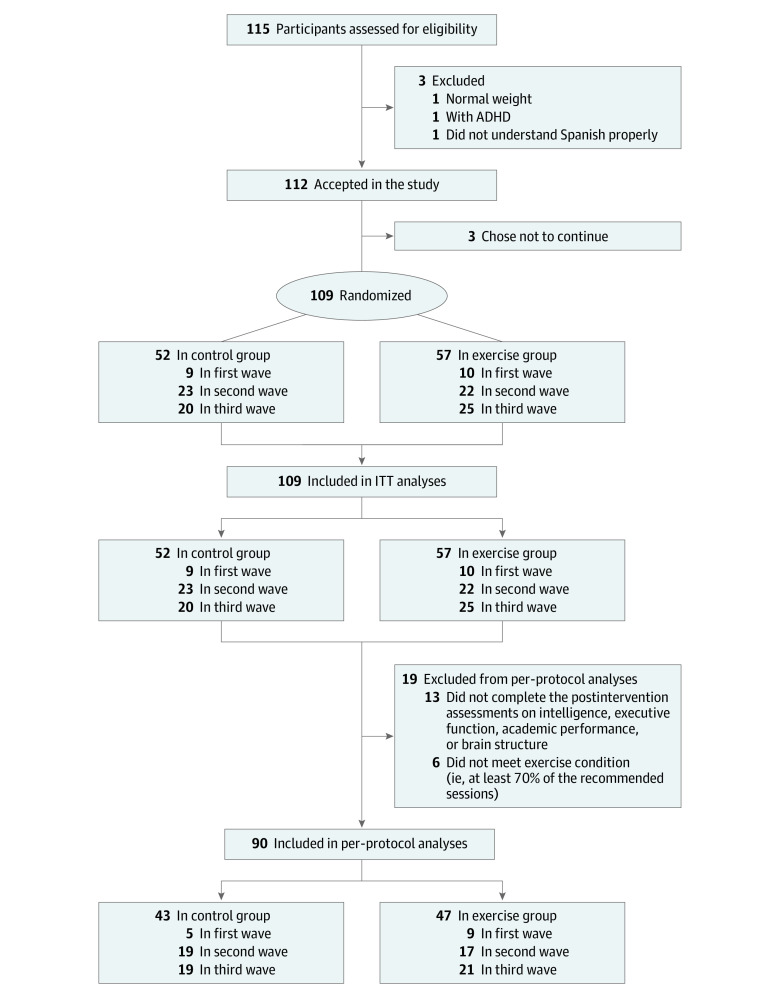
CONSORT Flow Diagram For final intention-to-treat (ITT) analyses, participants who left the study during the intervention or who did not complete the postexercise program assessments were imputed (see Statistical Analysis section). The actual number for each variable can be seen in eTables 1 to 22 in Supplement 2. ADHD indicates attention-deficit/hyperactivity disorder.

The ActiveBrains trial^26^ is a parallel-group RCT conducted among children aged 8 to 11 years with overweight or obesity. The recruitment occurred mainly at the pediatric units of the 2 main hospitals in Granada, Spain. A total of 109 participants were randomly assigned (simple randomization conducted with SPSS, version 25.0 [IBM Corp]) to a control group or an exercise group. The flowchart of the study is presented in Figure 1. All preexercise and postexercise data were collected from November 21, 2014, to June 30, 2016. The parents or legal guardians of the children provided written informed consent to participate in the trial. The ActiveBrains project was approved by the ethics committee of the University of Granada, and it was registered on ClinicalTrials.gov (NCT02295072). This trial followed the Consolidated Standards of Reporting Trials (CONSORT) reporting guideline.

### Power and Sample Size

Our study was powered to detect small- to medium-sized effects (ie, Cohen *d* = 0.3), with an α error of 5% and a power of 80% with the inclusion of 90 participants. After adjustement for an estimated 10% estimated dropout rate (a similar rate has been observed in previous trials^43^), 100 participants were needed for sufficient power.

### Intervention and Control

The participants in the control group continued their usual routines. Both the control and exercise groups were provided with information about healthy nutrition and recommendations for physical activity at the beginning of the study. The exercise group was instructed to attend at least 3 (of 5 offered) supervised exercise sessions per week. Sessions lasted 90 minutes (60 minutes of aerobic exercises plus 30 minutes of resistance exercises). To increase motivation and adherence, exercise sessions were based on games and playful activities that involved coordinative exercises.

### Outcome Measurements

#### Intelligence, Executive Function, and Academic Performance

All outcomes were assessed before and after the intervention. Crystallized intelligence, fluid intelligence, and total (ie, crystallized plus fluid) intelligence were assessed by the Spanish version of the Kaufman Brief Intelligence Test.^44^ Cognitive flexibility was assessed using the Design Fluency Test and the Trail Making Test. Inhibition was evaluated with a modified version of the Stroop Color-Word Test (paper-pencil version).^45,46,47^ Working memory was measured by a modified version of the Delayed Non-Match-to-Sample computerized task.^48^ Academic performance was assessed by the Spanish version of the Woodcock-Johnson III Tests of Achievement.^49^

#### Brain MRI Outcomes

The structural and functional MRI outcomes studied are summarized in eFigure 1 in Supplement 2. The MRI acquisition and the specific processing steps for each analysis are individually detailed in the eMethods in Supplement 2.

#### Cardiorespiratory Fitness, Biological Maturation, and Socioeconomic Status

Cardiorespiratory fitness was evaluated using a gas analyzer (General Electric Corp) while the participant was performing a maximal incremental treadmill test (ergometer; h/p/cosmos sports & medical gmbh).^43^ Peak height velocity, a common indicator of maturity in children and adolescents,^50^ was calculated through the equations of Moore et al.^51^ Parents self-reported their highest educational level attained and current occupation, as described elsewhere.^26,52^

### Overall Physical Activity Assessment Before and During the Intervention

Activity patterns at baseline and during the intervention (week 10) were assessed with hip- and wrist-worn accelerometers (GT3X+; ActiGraph LLC), as described elsewhere.^53^

### Statistical Analysis

Neuroimaging data processing and analyses were conducted from June 1, 2017, to December 20, 2021. We report the findings from the per-protocol analyses in the main article and the intention-to-treat analyses in the eAppendix and eTables 19 to 21 in Supplement 2 based on 2 reasons: (1) we aimed to study the efficacy of the program rather than its effectiveness, and (2) in neuroimaging, it is technically difficult to apply imputation methods on images, and rarely done. The analyses of the effects of the intervention were tested using analysis of covariance, with behavioral outcomes and several MRI outcomes (hippocampal volume as the primary region of interest) as dependent variables in separate models, group (exercise vs control) as a fixed factor, and the baseline of the study outcome as a covariate. The intervention effects are presented as *z* scores of change, indicating that the SDs of the postexercise program values changed from the baseline mean and SD values (ie, the standardized effect size of the change^54^). This effect size can be interpreted according to the standard benchmarks (ie, approximately 0.2 SDs is considered a small effect size, approximately 0.5 SDs is considered a medium effect size, and approximately 0.8 SDs is considered a large effect size).^55^ Results in the raw units of measure are also provided in eTables 1 to 22 in Supplement 2. All *P* values were from 2-sided tests and results were deemed statistically significant at *P* < .05. In addition, we applied multiple testing corrections on the primary outcomes following the false discovery rate method proposed by Benjamini and Hochberg.^56^ A posteriori–planned analyses consisted of exploring potential mediators and moderators. Our mediation analyses are in line with the A Guideline for Reporting Mediation Analyses (AGReMA) statement. The statistical procedures were performed using SPSS software, version 25.0 (IBM Corporation) and R software, version 3.1.2 (R Group for Statistical Computing).

## Results

The baseline characteristics of the participants are presented in eTable 1 in Supplement 2. Of the 109 randomized participants (45 girls [41.3%]; mean [SD] body mass index [calculated as weight in kilograms divided by height in meters squared] of 26.8 [3.6] and mean [SD] age of 10.0 [1.1] years at baseline), 96 completed the trial (11.9% attrition rate), and 90 met the criteria for the per-protocol analyses (82.6% of the original sample). A graphical illustration of the a priori–planned and a posteriori–planned analyses of brain health outcomes is presented in eFigure 1 in Supplement 2. Additional details are provided in the eAppendix in Supplement 2.

### A Priori–Planned Analyses

**Figure 2.**
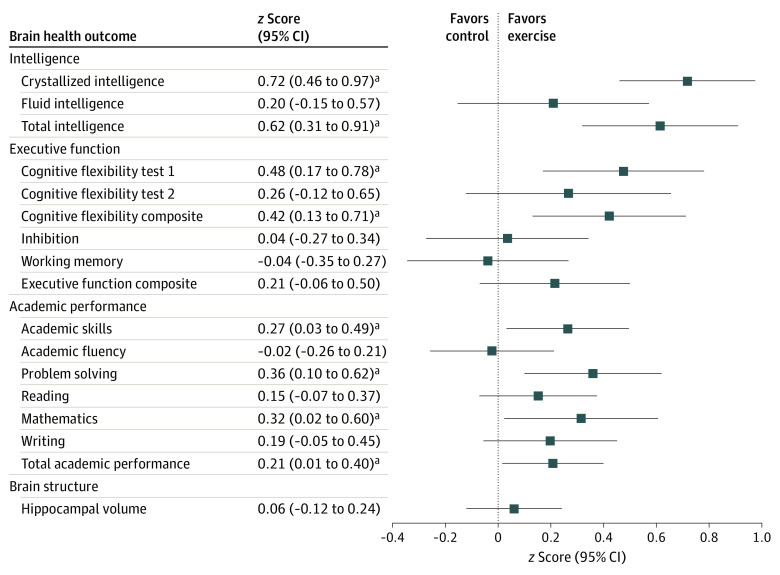
Per-Protocol Effects of the ActiveBrains Exercise Program on the Main Brain Health Outcomes Dots indicate the between-groups difference in *z* scores of change (ie, postexercise outcomes with respect to the baseline mean [SD] value). Bars indicate 95% CIs. Each analysis was adjusted for baseline outcomes. The cognitive flexibility composite *z* score was calculated as the renormalized mean of the *z* scores for cognitive flexibility test 1 and cognitive flexibility test 2. The executive function composite *z* score was calculated as the renormalized mean of the *z* scores for cognitive flexibility, inhibition, and working memory. Academic skills are the sum of components based on basic skills, such as reading decoding, mathematics calculation, and spelling. Academic fluency is the sum of tests based on reading, calculation, and writing fluency. Problem solving is the sum of the components based on solving academic problems in reading, mathematics, and writing. Total academic performance is the overall measure of academic performance based on reading, mathematics, and writing. Two of the cognitive tests (ie, the cognitive flexibility test 2 [Trail Making Test] and the inhibition test [Stroop Color-Word Test]) were originally expressed inversely, which means that lower scores indicate better performance. To simplify the visual interpretation of the main findings, we inverted these 2 scores so that they can be interpreted in the same fashion as the rest of the outcomes (ie, higher score indicates better performance). These cognitive tests are expressed in their original units and not inverted in eTables 2 and 19 in Supplement 2. ^a^Significant effect at *P* < .05 (or by the 95% CI not including zero).

The a priori–planned analyses included the effects of the exercise intervention on intelligence, executive function, academic performance, and hippocampal volume. The largest effect size observed in the ActiveBrains exercise program was for crystallized intelligence, with the exercise group improving from before exercise to after exercise (mean *z* score, 0.62 [95% CI, 0.44-0.80]) compared with the control group (mean *z* score, –0.10 [95% CI, –0.28 to 0.09]; difference between groups, 0.72 SDs [95% CI, 0.46-0.97]; *P* < .001) (Figure 2; eTable 2 in Supplement 2). Total intelligence also improved significantly more among the exercise group (mean *z* score, 0.69 [95% CI, 0.48-0.89]) than among the control group (mean *z* score, 0.07 [95% CI, –0.14 to 0.28]; difference between groups, 0.62 SDs [95% CI, 0.31-0.91]; *P* < .001). In addition, exercise positively affected a composite score of cognitive flexibility, derived from 2 cognitive flexibility tests (mean *z* score: exercise group, 0.25 [95% CI, 0.05-0.44]; control group, –0.17 [95% CI, –0.39 to 0.04]; difference between groups, 0.42 SDs [95% CI, 0.13-0.71]; *P* = .005). Within this composite, the largest improvement was observed for performance on cognitive flexibility test 1 (ie, the Design Fluency Test) (mean *z* score: exercise group, 0.65 [95% CI, 0.44-0.86]; control group, 0.18 [95% CI, –0.04 to 0.39]; difference between groups, 0.48 SDs [95% CI, 0.17-0.78]; *P* = .003). The exercise program had a null effect on inhibition (mean *z* score: exercise group, –0.51 [95% CI, –0.72 to –0.30]; control group, –0.48 [95% CI, –0.70 to –0.25]; difference between groups, 0.04 SDs [95% CI, –0.27 to 0.34]; *P* = .82) and working memory (mean *z* score: exercise group, 0.01 [95% CI, –0.20 to 0.22]; control group, 0.05 [95% CI, –0.17 to 0.27]; difference between groups, –0.04 SDs [95% CI, –0.35 to 0.27]; *P* = .80).

For academic performance, exercise improved total academic performance (mean *z* score: exercise group, 0.31 [95% CI, 0.18-0.44]; control group, 0.10 [95% CI, –0.04 to 0.24]; difference between groups, 0.21 SDs [95% CI, 0.01-0.40]; *P* = .03) and, particularly, mathematics (mean *z* score: exercise group, 0.35 [95% CI, 0.15-0.55]; control group, 0.04 [95% CI, –0.17 to 0.25]; difference between groups, 0.32 SDs [95% CI, 0.02-0.60]; *P* = .04), problem solving (mean *z* score: exercise group, 0.41 [95% CI, 0.24-0.59]; control group, 0.05 [95% CI, –0.13 to 0.24]; difference between groups, 0.36 SDs [95% CI, 0.10-0.62]; *P* = .007), and academic skills (mean *z* score: exercise group, 0.27 [95% CI, 0.11-0.43]; control group, 0.01 [95% CI, –0.16 to 0.17]; difference between groups, 0.27 SDs [95% CI, 0.03-0.49]; *P* = .03) (Figure 2; eTable 3 in Supplement 2). The exercise program had a small, nonsignificant effect on reading and writing skills and a null effect on academic fluency. In exploratory analyses, the positive effect of exercise on total academic performance, mathematics, and academic skills was mediated (30%-39% of mediation) by exercise-induced improvements in cognitive flexibility (eFigure 2A-C in Supplement 2). The improvements in academic problem solving were mediated (15% of mediation) by exercise-induced improvements in fluid intelligence (eFigure 2D in Supplement 2). However, the exercise program did not have an effect on overall hippocampal volume (mean *z* score: exercise group, 0.19 [95% CI, 0.07-0.32]; control group, 0.13 [95% CI, 0.00-0.27]; difference between groups, 0.06 SDs [95% CI, –0.12 to 0.24]; *P* = .50; Figure 2; eTable 4 in Supplement 2).

After correction for multiple comparisons of the primary outcomes (the 17 outcomes shown in Figure 2), the larger effects on crystallized intelligence (mean *z* score, 0.72 [95% CI, 0.46-0.97]; *P* ≤ .001), total intelligence (mean *z* score, 0.62 [95% CI, 0.31-0.91]; *P* ≤ .001), and the cognitive flexibility composite (mean *z* score, 0.42 [95% CI, 0.13-0.71]; *P* = .02) persisted. Likewise, the effects on problem solving continued to be significant (mean *z* score, 0.36 [95% CI, 0.10-0.62]; corrected *P* = .02), whereas the effects became nonsignificant for mathematics (mean *z* score, 0.32 [95% CI, 0.02-0.60]; corrected *P* = .07), academic skills (mean *z* score, 0.27 [95% CI, 0.03-0.49]; corrected *P* = .07), and total academic performance (mean *z* score, 0.21 [95% CI, 0.01-0.40]; corrected *P* = .07).

### A Posteriori–Planned Analyses of Brain MRI Outcomes

As shown in eFigure 1 in Supplement 2, we explored the effects of the intervention on a set of brain MRI outcomes, including volumetric analyses of hippocampus subregions and the prefrontal cortex (eTables 4-5 in Supplement 2); the cortical thickness, surface area, and subregions of the prefrontal cortex (eTables 6-7 in Supplement 2); and the functional connectivity between the hippocampus and prefrontal cortex (eTables 8-13 in Supplement 2). We also studied the effects of the intervention using a broader brain approach, including gray matter volumes of subcortical brain structures (eTable 14 in Supplement 2), morphologic (shape) analysis of subcortical brain structures (eFigure 3 in Supplement 2), total brain volumes (eTable 15 in Supplement 2), whole-brain voxelwise volumetric analysis, and whole-brain structural covariance network analysis (eFigure 4, eTable 16 in Supplement 2). Our intervention did not have a significant effect on any of these MRI outcomes.

### Effects of the Intervention on CRF and Its Role as Mediator

**Figure 3.**
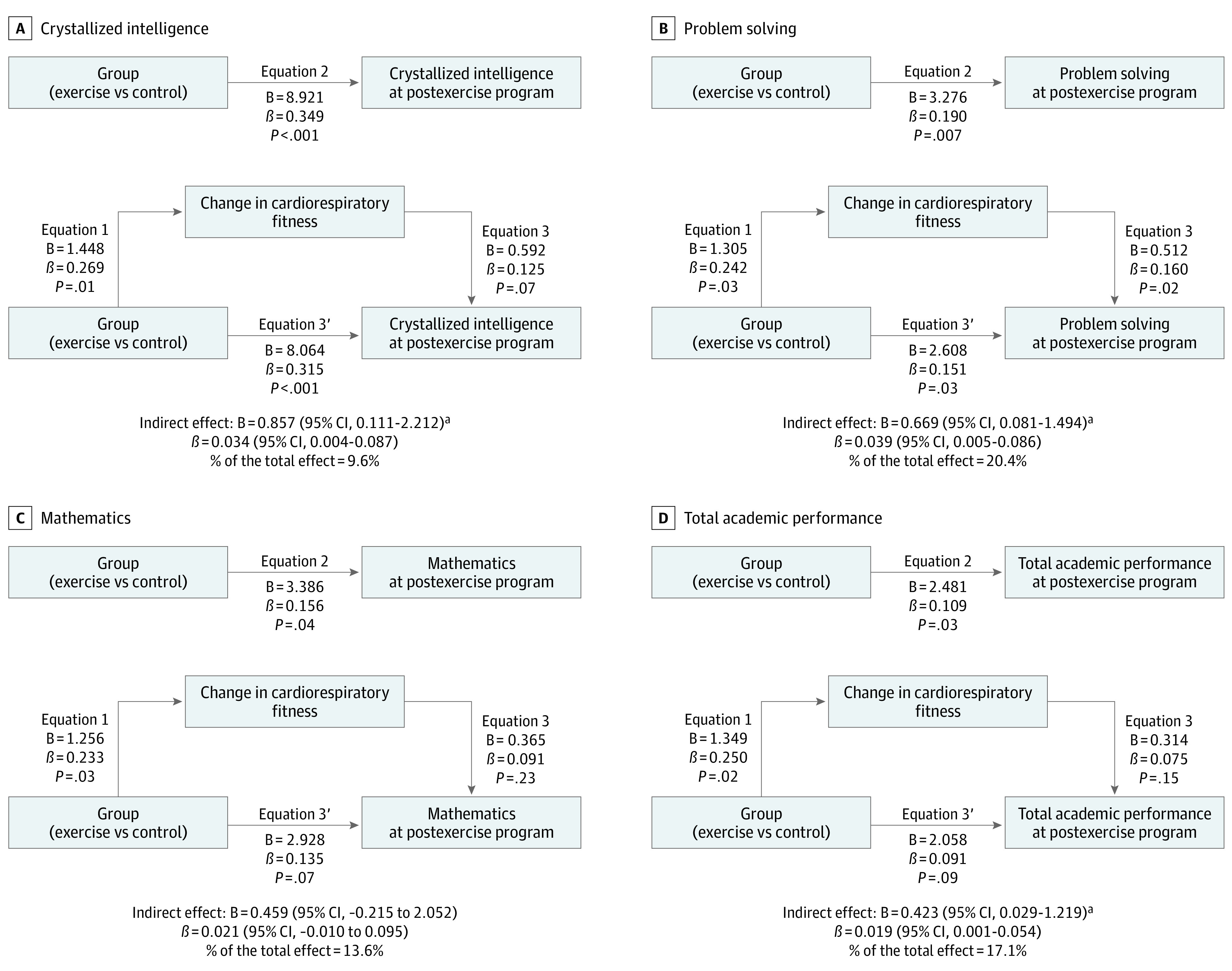
Cardiorespiratory Fitness Change Mediation Models of the Intervention Effects (ie, Exercise vs Control) on Crystallized Intelligence and Academic Performance Outcomes in Children With Overweight or Obesity Each analysis was adjusted by the respective intelligence or academic performance outcomes at baseline. Change in cardiorespiratory fitness expresses the change in total completion time (minutes) of the treadmill test at postexercise program with respect to the total completion time (minutes) at baseline because it was the main cardiorespiratory fitness outcome influenced by the exercise program. Problem solving is the sum of the components based on solving academic problems in reading, mathematics, and writing. Total academic performance is the overall measure of the academic performance based on reading, mathematics, and writing. B indicates unstandardized regression coefficient; β, standardized regression coefficient. ^a^Significant indirect effect at *P* < .05.

The exercise program improved CRF as indicated by treadmill time to exhaustion (mean *z* score: exercise group, 0.54 [95% CI, 0.27-0.82]; control group, 0.13 [95% CI, –0.16 to 0.41]; difference between groups, 0.42 SDs [95% CI, 0.01-0.82]; *P* = .04) (eTable 17 in Supplement 2). A consistent improvement, although smaller and nonsignificant, was observed in peak oxygen consumption, expressed in milliliters per kilogram per minute (mean *z* score: exercise group, 0.39 [95% CI, 0.13-0.65]; control group, 0.10 [95% CI, –0.18 to 0.37]; difference between groups, 0.29 SDs [95% CI, –0.08 to 0.67]; *P* = .13). The effects of the exercise program on crystallized intelligence, problem solving, and total academic performance were significantly mediated by improvements in CRF (ie, time to exhaustion), with a mediation effect of 10% to 20% (Figure 3).

### Moderators of the Intervention Effects

**Figure 4.**
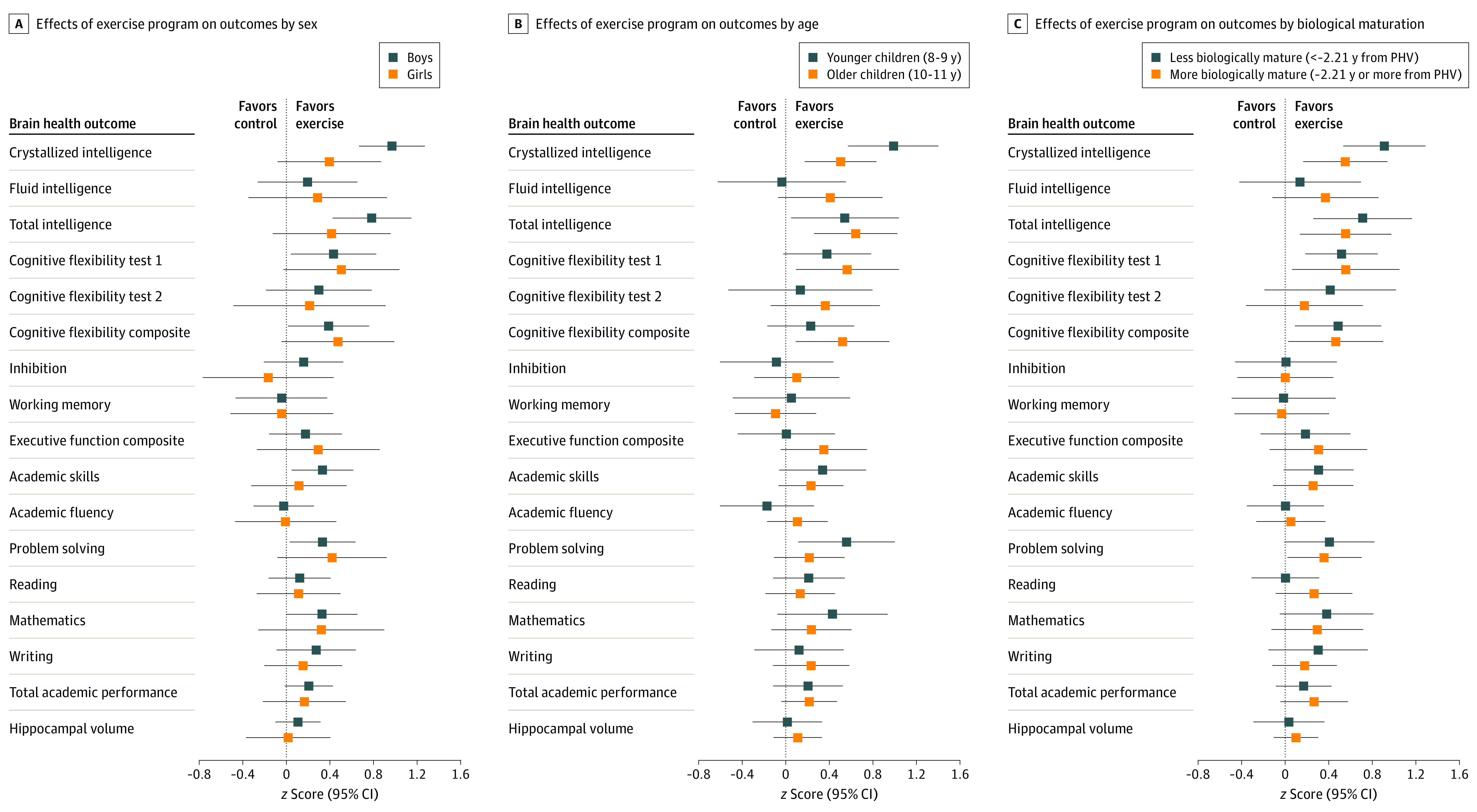
Per-Protocol Effects of the ActiveBrains Exercise Program on the Main Brain Health Outcomes by Sex, Age, and Biological Maturation Each analysis was adjusted by baseline outcomes. Dots indicate the between-groups difference in *z* scores of change (ie, postexercise outcomes with respect to the baseline mean [SD] value). Bars indicate 95% CIs. To express biological maturation, the number of years from peak height velocity (PHV) was calculated by subtracting the age of PHV from the chronological age. The difference in years was used as a measure of maturity. Peak height velocity was dichotomized using the median. The cognitive flexibility composite *z* score was calculated as the renormalized mean of the *z* scores for cognitive flexibility test 1 and cognitive flexibility test 2. Executive function composite *z* score was calculated as the renormalized mean of the *z* scores for cognitive flexibility, inhibition, and working memory. Academic skills are the sum of components based on basic skills such as reading decoding, mathematics calculation, and spelling. Academic fluency is the sum of tests based on reading, calculation, and writing fluency. Problem solving is the sum of the components based on solving academic problems in reading, mathematics, and writing. Total academic performance is the overall measure of academic performance based on reading, mathematics, and writing. Two of the cognitive tests (ie, cognitive flexibility test 2 [Trail Making Test] and the inhibition test [Stroop Color-Word Test]) were originally expressed inversely, which means that lower scores indicate better performance. To simplify the visual interpretation of the main findings, we inverted these 2 scores so that they can be interpreted in the same fashion as the rest of the outcomes (ie, higher score indicates better performance). These cognitive tests are expressed in their original units and not inverted in eTables 2 and 19 in Supplement 2.

Figure 4 shows that the effect sizes of the exercise program were consistent across sex, age, and maturation for most of the primary outcomes studied, except for crystallized intelligence, for which the exercise program was more effective for boys, younger participants, and less mature participants. The sex differences observed could be partially explained by the finding that boys spent more time at high-intensity zones (ie, over their individualized anaerobic threshold monitored with heart rate) (eTable 18 in Supplement 2). We also observed that children with lower socioeconomic status showed larger improvements in fluid and total intelligence, as did children with a lower performance at baseline on the intelligence test (eFigure 5 in Supplement 2).

### Exploratory Analyses Related to the Interpretation of the Intervention Effects

#### Intention-to-Treat and Dropout Analyses

The main effects of this intervention observed on intelligence and cognitive flexibility remained significant in intention-to-treat analyses (eTables 19-21 in Supplement 2), indicating the robustness of the main findings (further details in the eAppendix in Supplement 2). Participants who withdrew during the trial did not differ from those completing the study in any of the behavioral outcomes studied (eTable 22 in Supplement 2).

#### Compensatory and Contamination Effects

**Figure 5.**
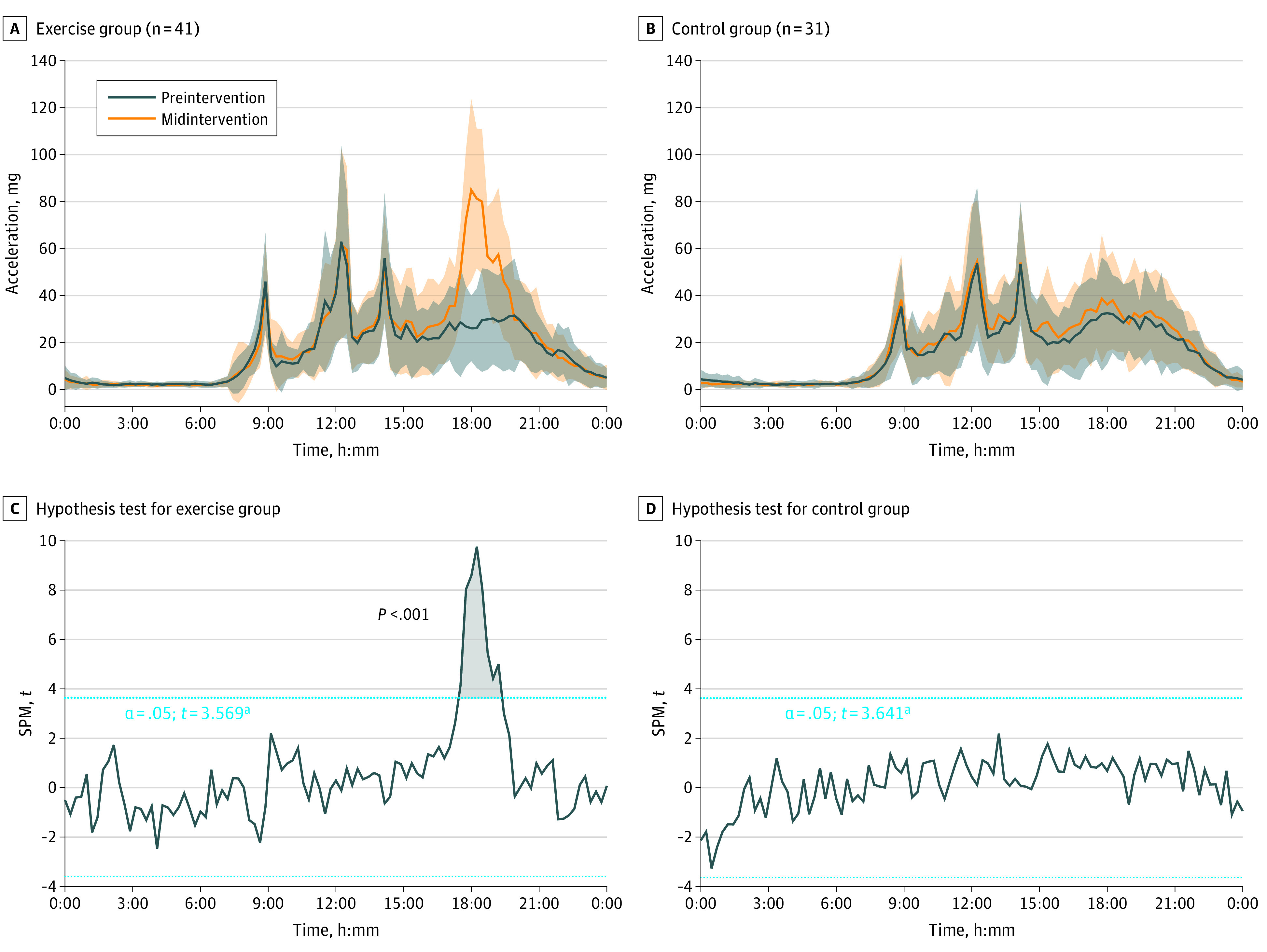
Comparison of the 24-Hour Physical Activity Patterns Derived From Aggregated Raw Accelerations Measured With an Accelerometer Attached at the Right Hip at Baseline and in the Middle of the Exercise Program SPM indicates statistical parametric mapping. ^a^The hypothesis test shows the threshold at which there are significant differences in physical activity patterns between the baseline and exercise periods.

The children in the exercise group significantly increased their activity levels during the time of day in which they were participating in the exercise program, without reductions (ie, no compensation) during other times of the day (results from the hip-attached accelerometer in Figure 5; results from the wrist-attached accelerometer in eFigure 6 in Supplement 2). The children in the control group kept the same levels of daily activity (ie, no contamination).

#### Volume and Intensity of the Exercise Program

We observed a mean (SD) heart rate intensity of 138 (8) beats per minute per session, indicating that the children trained for more than 1 hour at 70% of their maximum heart rate. The children accumulated, on average, 38% of the session time (ie, 25 minutes) at high intensities above 80% of their maximum heart rate (eFigure 7 in Supplement 2). The distribution of the attendance to the exercise sessions is presented in eFigure 8 in Supplement 2.

## Discussion

### Overview of the Main Findings

The ActiveBrains trial contributes to the existing literature with several novel findings. First, a 20-week aerobic and resistance exercise program including coordinative exercises, performed at relatively high intensity for more than 1 hour, 3 times per week, improved total and crystallized intelligence, cognitive flexibility, and academic performance among children with overweight or obesity. We rely mainly on the observed effects on intelligence, particularly on crystallized intelligence, as well as on cognitive flexibility, given the effect sizes and significance observed.^57^ In fact, the effects on intelligence and cognitive flexibility outcomes were consistent and robust, persisting after applying multiple testing corrections to the per-protocol and intention-to-treat analyses. However, the exercise program had a null effect on other executive functions, such as inhibition and working memory, as well as on hippocampal volume. Second, we did not observe any significant effects of exercise on the brain MRI outcomes studied (a posteriori–planned analyses) and therefore could not investigate whether changes in brain structure or function mediated the effects observed on behavioral outcomes. Third, the effects of the exercise program on crystallized intelligence, total academic performance, and problem solving were partially mediated by exercise-induced improvements in CRF (10%-20%; small mediation effect). Improvements in most academic performance indicators were largely mediated (approximately 30%-39% of mediation) by exercise-induced changes in cognitive flexibility. Fourth, the exercise effects were rather consistent across sex, age, socioeconomic status, and baseline level subgroups for most of the study outcomes, except for intelligence outcomes that improved more for boys than for girls. The interpretation of the results should be made in conjunction with the characteristics of the exercise intervention. The eAppenix in Supplement 2 includes an extended discussion on: (1) the potential compensatory or contamination effects, (2) the combination of aerobic and resistance training that additionally included a coordinative component and cognitive demands, (3) a thorough analysis of the intensity of the exercise program, and (4) an interpretation of the different cognitive flexibility tests used in this study and the mediators and moderators of the main exercise effects (secondary analyses).

### Findings in the Context of Previous Studies

To our knowledge, only 3 previous intervention studies have tested the long-term effects of exercise on intelligence in a pediatric population. The first study tested the effects of a yoga program but did not include a control group.^58^ The second study, a cluster school–based RCT, investigated the effects of daily physical education sessions, yet half of the “control” group also received daily physical education for half of the intervention period.^59^ The third study was a school-based pilot study conducted by our group among only 17 to 20 children per study group, which investigated the effects of increasing the intensity and the number of physical education sessions per week.^60^ The conclusions from these 3 studies suggest the potential benefits of exercise. Given the preliminary nature of these findings and the limitations associated with the study design and sample size, the ActiveBrains RCT provides the strongest evidence thus far regarding a causal effect of physical exercise on intelligence, particularly crystallized intelligence, which is denoted by a large effect (ie, ≥9 points in the typical punctuation of the test, equivalent to 0.7 SDs, with larger improvements in the exercise group). Although previous evidence for the long-term effects of exercise on intelligence is limited, more evidence is available for the short-term effects of exercise.^61^ The 2018 Physical Activity Guidelines Scientific Advisory Report concluded that there is evidence supporting an improvement in crystallized intelligence in children after a single bout of moderate-to-vigorous physical activity,^8,61^ which supports our findings.

Our exercise program demonstrated a medium-sized effect on cognitive flexibility and null effects for the other executive functions tested. Systematic reviews and meta-analyses of children and adolescents have reported a significant effect of exercise on overall executive function,^62,63,64,65,66^ with mixed conclusions among reviews when referring to the specific dimensions of this complex cognitive construct. The diversity of cognitive tasks used and the different characteristics of the exercise interventions (ie, mode, frequency, duration of session, intensity, and length of intervention) across studies might explain the discrepancies among the individual studies. However, the recently synthesized cumulative evidence supports a positive effect of exercise on the 3 core executive functions: working memory, inhibition, and cognitive flexibility.^66^

Our findings are in line with existing literature concerning academic performance, in which exercise has specifically improved mathematics to a higher extent than other academic subjects, including language.^67,68^ In our study, the positive effect of exercise on mathematics was partly explained by exercise-induced improvements in fluid intelligence, and the positive effect of exercise on total academic performance, problem solving, and academic skills was partly mediated by exercise-induced improvements in cognitive flexibility. These findings suggest that this particular executive function plays an important role in academic performance^69,70,71^ and contributes to our understanding of the cognitive processes by which exercise improves academic performance.

Our exercise program had no significant effects on any of the MRI outcomes studied. Further discussion on whether the intervention length or sample size could have influenced these null findings is in the eAppendix in Supplement 2. Previous studies (4 trials conducted in the US and 1 in Canada) conducted among children observed positive effects of exercise on white matter integrity,^12,14,20^ task-based functional MRI findings,^16,17,18^ and resting-state synchrony.^15^ We believe that some brain outcomes must have changed in our participants in the exercise group to explain the observed changes in intelligence and cognitive flexibility. Those changes change could have occurred at a molecular or cellular level or could have been due to some other features that were undetected with the neuroimaging techniques used herein. The continuous advances in the neuroimaging field will open new avenues for the study of the effects of exercise on the human brain.

### Limitations

This study has some limitations. It is unknown whether longer interventions are needed to elicit structural or functional changes in the brain (eAppendix in Supplement 2). Furthermore, although several protocols were adopted to reduce the risk of bias in the evaluations (eg, randomization after baseline assessment and the use of physical trainers not involved in the evaluations), some of the project staff involved in the postexercise evaluations were not blinded to the group allocation for practical reasons. Even assuming an attenuation of the effect sizes after correcting for potential bias, we believe that the main exercise effects on intelligence and cognitive flexibility would remain significant given their magnitude, making an attenuation of the effect size unlikely to change the study conclusions. Additionally, the extent to which the findings from our study conducted among children with overweight or obesity applies to other populations is unknown.

## Conclusions

The findings of this RCT support that intelligence and cognitive flexibility are improved after 20 weeks of exercise of relatively high intensity for more than 1 hour, 3 times per week, and during a sensitive period of life (ie, childhood) when the brain is growing and developing. We failed to detect which structural or functional changes in the brain may underlie these exercise effects on behavioral outcomes. We also observed that exercise-induced changes in CRF explain some of the exercise benefits, although not most of them. Moreover, our exercise program had small effects on academic performance indicators (ie, mathematics, problem solving, and total academic performance) that were mediated by exercise-induced improvements in cognitive flexibility and fluid intelligence; these effects were consistent with those described in the existing literature. Finally, the intervention effects were generally consistent across the moderators studied, except for larger improvements in intelligence outcomes among boys compared with girls. This trial provides a comprehensive investigation of the effects of exercise on cognitive outcomes and academic performance during childhood in the presence of overweight or obesity. However, the brain mechanisms underlying those effects remain unknown.

## Notes

Supplement 1.
Trial Protocol and Statistical Analysis Plan

Supplement 2.
eMethods.

eAppendix.

eReferences.
**eFigure 1.** Graphical Illustration of the a Priori Planned Main Analyses of the Study, as Well as the a Posteriori Planned Exploratory Analyses Conducted on Different Brain Health Outcomes **eFigure 2.** Cognitive Flexibility and Fluid Intelligence Mediation Models of the Intervention Effects (ie, Exercise vs Control) on Academic Performance Outcomes in Children With Overweight or Obesity **eFigure 3.** An Illustration of the Shape Analysis of Subcortical Brain Structures **eFigure 4.** Structural Covariance Networks Delineated by Non-Negative Matrix Factorization Analysis **eFigure 5.** Per-Protocol Effects of the ActiveBrains Exercise Program on the Main Brain Health Outcomes by Parental Educational Levels (A), Parental Occupational Levels (B), and Baseline Levels (C) **eFigure 6.** Comparison of the 24 h Physical Activity Patterns Derived From Aggregated Raw Accelerations (ie, Euclidean Norm Minus One Accelerations) Measured With an Accelerometer Attached at the Nondominant Wrist at Baseline (ie, Black Line) and in the Middle of the Exercise Program (ie, Orange Line) in Exercise and Control Groups **eFigure 7.** Violin Plots Characterizing the Intensity of the Exercise Program as Measured by Heart Rate (HR) Monitors **eFigure 8.** Box Plot Showing the Distribution of the Attendance to the Exercise Program **eTable 1.** Descriptive Baseline Characteristics of the ActiveBrains Participants Meeting Intention-to-Treat Criteria **eTable 2.** Per-Protocol Effects of the ActiveBrains Exercise Program on Raw and *z*-Score Post-Exercise (ie, *z*-Score of Change From Baseline) Intelligence and Executive Function Outcomes **eTable 3.** Per-Protocol Effects of the ActiveBrains Exercise Program on Raw (Standard Score) and *z*-Score Post-Exercise (*z*-Score of Change From Baseline) Academic Performance Outcomes (Woodcock-Muñoz Standardized Test) **eTable 4.** Per-Protocol Effects of the ActiveBrains Exercise Program on Raw (mm^3^) and *z*-Scores of Post-Exercise Hippocampal Volume **eTable 5.** Per-Protocol Effects of the ActiveBrains Exercise Program on Raw (mm^3^) and *z*-Scores of Post-Exercise Prefrontal Cortex Gray Matter Volume Outcomes **eTable 6.** Per-Protocol Effects of the ActiveBrains Exercise Program on Raw (mm) and *z*-Scores of Post-Exercise Prefrontal Cortex Cortical Thickness Outcomes **eTable 7.** Per-Protocol Effects of the ActiveBrains Exercise Program on Raw (mm^2^) and *z*-Scores of Post-Exercise Prefrontal Cortex Surface Area Outcomes **eTable 8.** Per-Protocol Effects of the ActiveBrains Exercise Program on Raw (β Values) and *z*-Scores of Post-Exercise Left Hippocampal Functional Connectivity With Prefrontal Cortex Subregions **eTable 9.** Per-Protocol Effects of the ActiveBrains Exercise Program on Raw (β Values) and *z*-Scores of Post-Exercise Left Anterior Hippocampal Functional Connectivity With Prefrontal Cortex Subregions **eTable 10.** Per-Protocol Effects of the ActiveBrains Exercise Program on Raw (β Values) and *z*-Scores of Post-Exercise Left Posterior Hippocampal Functional Connectivity With Prefrontal Cortex Subregions **eTable 11.** Per-Protocol Effects of the ActiveBrains Exercise Program on Raw (β Values) and *z*-Scores of Post-Exercise Right Hippocampal Functional Connectivity With Prefrontal Cortex Subregions **eTable 12.** Per-Protocol Effects of the ActiveBrains Exercise Program on Raw (β Values) and *z*-Scores of Post-Exercise Right Anterior Hippocampal Functional Connectivity With Prefrontal Cortex Subregions **eTable 13.** Per-Protocol Effects of the ActiveBrains Exercise Program on Raw (β Values) and *z*-Scores of Post-Exercise Right Posterior Hippocampal Functional Connectivity With Prefrontal Cortex Subregions **eTable 14.** Per-Protocol Effects of the ActiveBrains Exercise Program on Raw (mm^3^) and *z*-Scores of Post-Exercise Subcortical Brain Volumes Other Than the Hippocampus **eTable 15.** Per-Protocol Effects of the ActiveBrains Exercise Program on Raw (cm^3^) and *z*-Scores of Post-Exercise Total Brain Volumes **eTable 16.** Per-Protocol Effects of the ActiveBrains Exercise Program on Raw (Loadings) and *z*-Scores of Post-Exercise Structural Covariance Network **eTable 17.** Per-Protocol Effects of the ActiveBrains Exercise Program on Raw (Loadings) and *z*-Scores of Post-Exercise Cardiorespiratory Fitness **eTable 18.** Sex Differences in Intensity Monitored by Heart Rate During the Exercise Sessions **eTable 19.** Intention-to-Treat Effects of the ActiveBrains Exercise Program on Raw and *z*-Scores of Post-Exercise Intelligence and Executive Function Outcomes **eTable 20.** Intention-to-Treat Effects of the ActiveBrains Exercise Program on Raw (Standard Score) and *z*-Scores of Post-Exercise Academic Performance Outcomes (Woodcock-Muñoz Standardized Test) **eTable 21.** Intention-to-Treat Effects of the ActiveBrains Exercise Program on Raw (mm^3^) and *z*-Scores of Post-Exercise Hippocampal Gray Matter Volume **eTable 22.** Descriptive Characteristics of the ActiveBrains Participants That Completed The Study (ie, Nondropouts) and Those That Did Not Complete The Study (ie, Dropouts) at Baseline

Supplement 3.
Data Sharing Statement
