# Supplementary material for: Effects of an Exercise Program on Brain Health Outcomes for Children With Overweight or Obesity: The ActiveBrains Randomized Clinical Trial
Source: JAMA Netw Open. 2022 Aug 30;5(8):e2227893. doi: 10.1001/jamanetworkopen.2022.27893 (PMC9428743; doi:10.1001/jamanetworkopen.2022.27893)
Supplement: Supplement 3. — Data Sharing Statement [file jamanetwopen-e2227893-s003.pdf]

Data Sharing Statement

Ortega. Effects of an Exercise Program on Brain Health Outcomes for Children With Overweight or Obesity. *JAMA Netw Open*. Published August 30, 2022. doi:10.1001/jamanetworkopen.2022.27893

Data
Data available: No

Additional Information
Explanation for why data not available: We did not obtain children's parents consent to widely share the data nor was it included in the IRB protocol.
